# Distribution and determinants of corneal volume among healthy young Chinese adults: a cross-sectional study

**DOI:** 10.1186/s12886-024-03342-8

**Published:** 2024-02-12

**Authors:** Gang Liang, Jia-Yan Kai, Dan-Lin Li, Zhi-Jian Yin, Yue-Zu Li, Rong Ma, Ya-Jie Zheng, Yu Qin, Chen-Wei Pan

**Affiliations:** 1 Department of Ophthalmology, the Affiliated Hospital of Yunnan University, Kunming, China; 2 Department of Ophthalmology, The Second People’s Hospital of Yunnan Province, Kunming, China; 3 School of Public Health, Suzhou Medical College of Soochow University, 199 Ren Ai Road, Suzhou, 215123 China; 4 Department of Ophthalmology, the First Affiliated Hospital of Dali University, Dali, China

**Keywords:** Corneal volume, Corneal thickness, Axial length, Keratometry, White-to-white

## Abstract

**Background:**

Several studies have previously reported the normal values of corneal volume (CV) in various populations, whereas little is known about the CV distribution in healthy young Chinese adults. Our study aimed to investigate the distribution of CV and its relationships with other ocular biometric parameters among healthy young Chinese adults.

**Methods:**

A total of 1645 eyes from 1645 students at Dali University in Yunnan Province, China, were analyzed. Pentacam was used to measure CV. Central corneal thickness (CCT) and biomechanically corrected intraocular pressure (bIOP) were evaluated by Corvis-ST. Other biometrical parameters, including axial length (AL), keratometry, and white-to-white (WTW) distance, were measured using IOL Master.

**Results:**

The mean age of the study population was 19.01 ± 0.92 years, and 68.81% of them were women. The CV was normally distributed in the whole sample, with a mean value of 61.23 ± 3.22 mm^3^. CV and CCT were significantly smaller in the Yi ethnic group than in the Han ethnic group (*p* < 0.01). CCT (coefficient: 0.085; *p* < 0.001) and keratometry (coefficient: 0.422; *p* < 0.001) were positively correlated with CV, while AL (coefficient: -0.204; *p* < 0.001), WTW distance (coefficient: -0.236; *p* < 0.001) and bIOP (coefficient: -0.06; *p* < 0.001) were inversely associated with CV.

**Conclusions:**

Our study provides an age-specific distribution of CV among healthy young Chinese adults. CCT, keratometry, AL, WTW distance and bIOP were important factors associated with CV.

**Supplementary Information:**

The online version contains supplementary material available at 10.1186/s12886-024-03342-8.

## Background

With the increasing application of refractive surgical procedures in recent years, corneal tomography, which characterizes the shape of the cornea, has become of critical importance in predicting postoperative outcomes [1]. Knowledge of the normal values of tomographic indices could provide valuable information for clinicians in screening surgery candidates.

Corneal volume (CV) is a useful tomographic parameter that reflects both the topographical and pachymetric properties of the cornea simultaneously [2]. Modern tomography systems such as Pentacam have enabled the calculation of CV in 3-, 5-, 7- and 10-mm diameter zones [3]. As it is a numerical value, it could be statistically analyzed to assess corneal integrity. In addition, the CV indirectly reflects the physiological function of the cornea [4]. An abnormal CV may give clues to corneal ectasia, corneal edema or other corneal disorders [5]. It has been proposed to be helpful not only in presurgical screening [6] but also in monitoring postoperative complications such as keratoconus and other ectatic changes [3]. Therefore, it is essential to have a normal range of CV to discriminate normal corneas from abnormal corneas.

The normal values of CV have been previously investigated in various populations [3–7]. However, little is known about the CV distribution in young Chinese adults, who are the largest population of refractive surgery candidates worldwide [8]. The aim of the present study was to investigate the distribution of CV and its determinants among healthy young Chinese adults.

## Methods

### Study design and participants

The participants of this cross-sectional study were students at Dali University in Yunnan Province, which is home to the largest ethnic group in China. All 2698 freshman entering the college in 2021 were initially invited. They were provided with a standardized questionnaire collecting demographic information (e.g. ethnicity) and medical histories. Exclusion criteria were (1) over 26 years old and (2) with eye disease (e.g., keratoconus, glaucoma, retinal detachment, and acute infection) or with laser corneal refractive surgery history. Ultimately, 1645 students were included for further analysis. Detailed information on the sampling procedures and quality control has been previously described [9].

#### Ocular examinations

The CVs of all participants were measured using the Pentacam tomographer (Oculus, Wetzlar, Germany), which could provide three-dimensional images of participants’ anterior segment with a rotating Scheimpflug camera [3]. According to the quality specification index provided by the Pentacam software, only images with good quality were considered valid. The CV contained within 10-mm diameter discs was calculated and used for analysis.

Axial length (AL), keratometry, anterior chamber depth (ACD), and white-to-white (WTW) distance were measured by IOL Master (Carl Zeiss Meditec AG, Jena, Germany). Central corneal thickness (CCT) and biomechanically corrected intraocular pressure (bIOP) were evaluated by Corvis-ST (CST, Oculus, Wetzlar, Germany). bIOP was adjusted for age, corneal thickness and other corneal biomechanical parameters and had improved accuracy. Refractive error was assessed by noncycloplegic refraction. Both eyes of each subject were examined, but only the data of the right eye were analyzed, as there was a high correlation of biometrical indices between the right and left eyes (Pearson correlation coefficient = 0.973). Each of the variables was measured twice for both eyes and the mean value was calculated and used in the analysis.

### Statistical analysis

Descriptive statistics are presented as the mean ± standard deviation (SD). Paired t tests and analysis of variance (ANOVA) were performed to make comparisons between different groups. The distribution of CV was evaluated by the Shapiro‒Wilk (SW) test. Locally weighted scatterplot smoothing (LOWESS) was utilized to explore the relationships between each ocular factor and CV. Smooth curves were fitted using local weighted regression to reveal the changing trend of data. Univariate and stepwise multivariate linear regression were both adopted to explore the associations between CV and other variables. The statistical significance level was set at 0.05. All statistical analyses were performed using SPSS 17.0 and Stata 16.0.

### Ethical issues

The present study was approved by the Ethics Committee of Affiliated Hospital of Yunnan University (approval number [No.2022-K050]) and was performed following the tenets of the Declaration of Helsinki. All participants were told of the brief contents of the research and gave informed consent before inclusion.

## Results

**Fig. 1:**
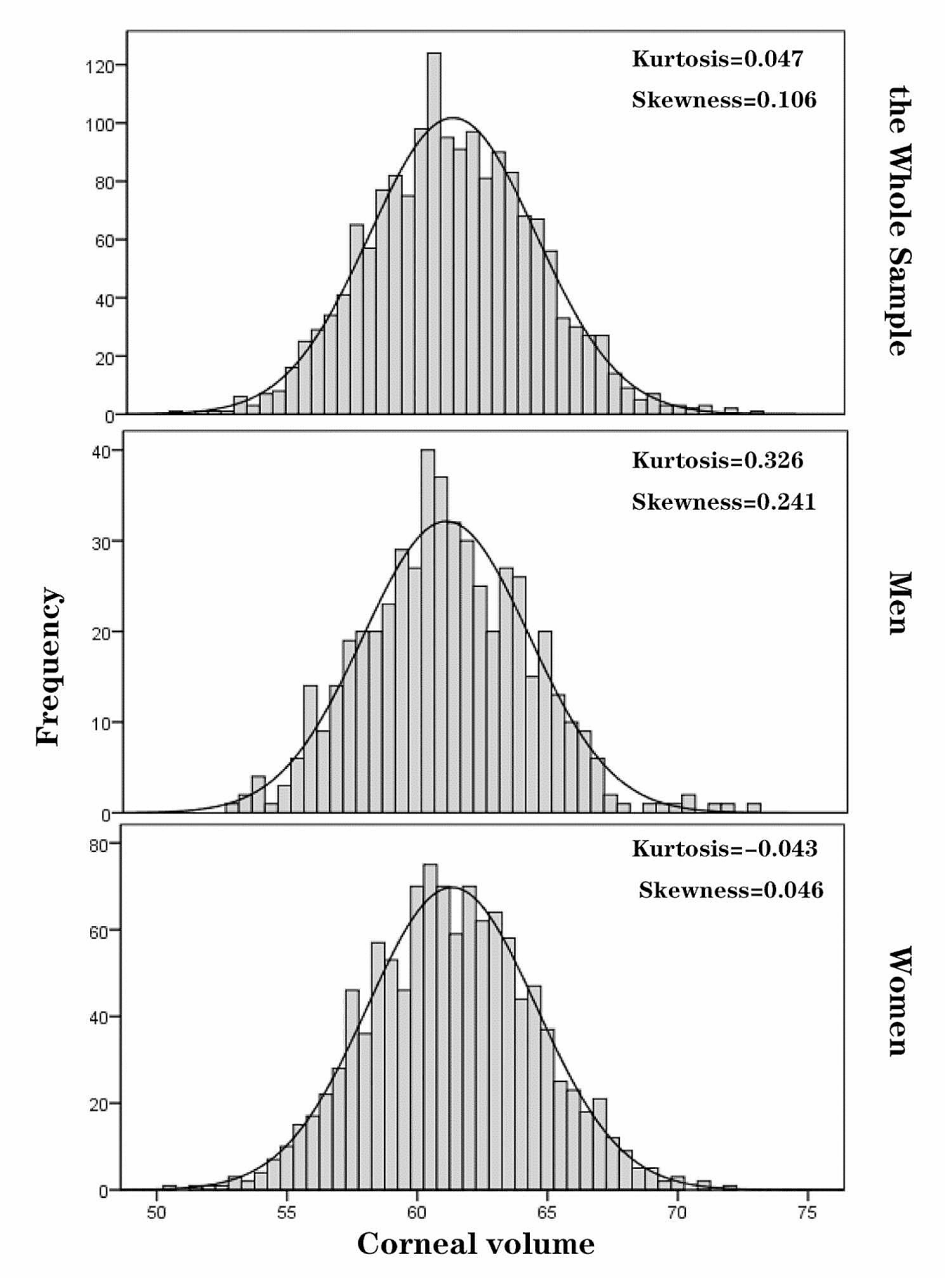
Distribution of corneal volume (mm^3^) in the whole sample and by sex

Among the 1645 included participants, 1132 (68.81%) were women, and the mean age was 19.01 ± 0.92 years. CV and other ocular biometrical properties of all subjects are summarized in Table 1. The mean CV in the whole sample was 61.23 ± 3.22 mm^3^. The mean CV was smaller in men (61.00 ± 3.18 mm^3^) than in women (61.33 ± 3.24 mm^3^), and the difference was statistically significant (*p* = 0.003). Figure 1 demonstrates the distribution of CV separately for men, women and all individuals. The CV was normally distributed in both women and the whole sample (SW *p* > 0.05), while the distribution of CV in men was slightly positively skewed (skewness = 0.326, kurtosis = 0.241, SW *p* = 0.02).

**Table 1:** General characteristics of the 1645 participants

	Total		Men, *n* = 513, 31.19%		Women, *n* = 1132, 68.81%		
Parameters	Mean ± SD	Range	Mean ± SD	Range	Mean ± SD	Range	*p*
Age, year	19.01 ± 0.92	15.69 to 24.41	19.06 ± 0.87	15.95 to 22.58	18.98 ± 0.95	15.69 to 24.41	0.02
CV, mm^3^	61.23 ± 3.22	50.50 to 72.80	61.00 ± 3.18	52.80 to 72.80	61.33 ± 3.24	50.50 to 71.90	0.003
AL, mm	24.85 ± 1.21	21.23 to 34.59	25.25 ± 1.28	21.23 to 34.29	24.67 ± 1.13	21.55 to 34.59	< 0.001
SE, D	-3.69 ± 2.37	-12.38 to + 1.38	-3.46 ± 2.49	-11.00 to + 1.00	-3.80 ± 2.31	-12.38 to + 1.38	< 0.001
Keratometry, diopter	42.91 ± 1.52	29.85 to 47.87	42.34 ± 1.58	29.85 to 46.23	43.17 ± 1.43	34.47 to 47.87	< 0.001
ACD, mm	3.62 ± 0.24	2.62 to 4.52	3.70 ± 0.25	2.62 to 4.52	3.59 ± 0.23	2.67 to 4.27	< 0.001
WTW, mm	12.10 ± 0.39	10.80 to 13.80	12.22 ± 0.37	11.00 to 13.20	12.04 ± 0.38	10.80 to 13.80	< 0.001
CCT, micron	542.78 ± 31.81	293.00 to 651.00	544.02 ± 31.93	422.00 to 651.00	542.22 ± 31.75	293.00 to 643.00	0.27
bIOP, mmHg	17.45 ± 3.03	10.50 to 51.50	17.17 ± 3.06	11.00 to 31.00	17.58 ± 3.01	10.50 to 51.50	0.001

Significance was tested using t tests

SD = standard deviation; CV = corneal volume; AL = axial length; SE = spherical equivalent; ACD = anterior chamber depth; WTW = white to white; CCT = central corneal thickness; bIOP = biomechanically corrected intraocular pressure

The distribution of CV stratified by ethnicity is shown in Supplemental Table S1. The Han, Yi, Nakhi, Bai, Dai, Zhuang and Miao ethnic groups were analyzed separately, whereas the others were combined as “other minorities”. Han and Yi adults accounted for 75.32% (*n* = 1239) and 9.18% (*n* = 151) of the whole sample, respectively. Although no significant difference in CV between ethnic groups was observed by ANOVA (F = 1.619, *p* = 0.12), the mean CV in the Yi ethnic group was significantly smaller than that in Han adults (*p* = 0.003). Furthermore, we compared other biometrical properties between Han and Yi adults in Table 2 and found that CCT was also significantly thinner in Yi adults (*p* = 0.004).

**Table 2:** Comparisons of ocular biometrical properties between the two major ethnic groups

Variable	Ethnic group		*p* value
	Han (*n* = 1239, 75.32%)	Yi (*n* = 151, 9.18%)	
CV, mm^3^	61.31 ± 3.24	60.49 ± 3.20	0.003*
AL, mm	24.85 ± 1.21	24.86 ± 1.03	0.93
SE, D	-3.72 ± 2.38	-3.56 ± 2.37	0.43
Keratometry, diopter	42.93 ± 1.56	42.76 ± 1.39	0.18
ACD, mm	3.63 ± 0.24	3.64 ± 0.24	0.47
WTW, mm	12.11 ± 0.94	12.11 ± 0.37	0.99
CCT, micron	543.54 ± 32.16	535.50 ± 31.53	0.004*
bIOP, mmHg	17.47 ± 3.01	17.19 ± 3.13	0.28

* *p* < 0.01; Significance was tested using t tests

CV = corneal volume; AL = axial length; SE = spherical equivalent; ACD = anterior chamber depth; WTW = white to white; CCT = central corneal thickness; bIOP = biomechanically corrected intraocular pressure

**Fig. 2:**
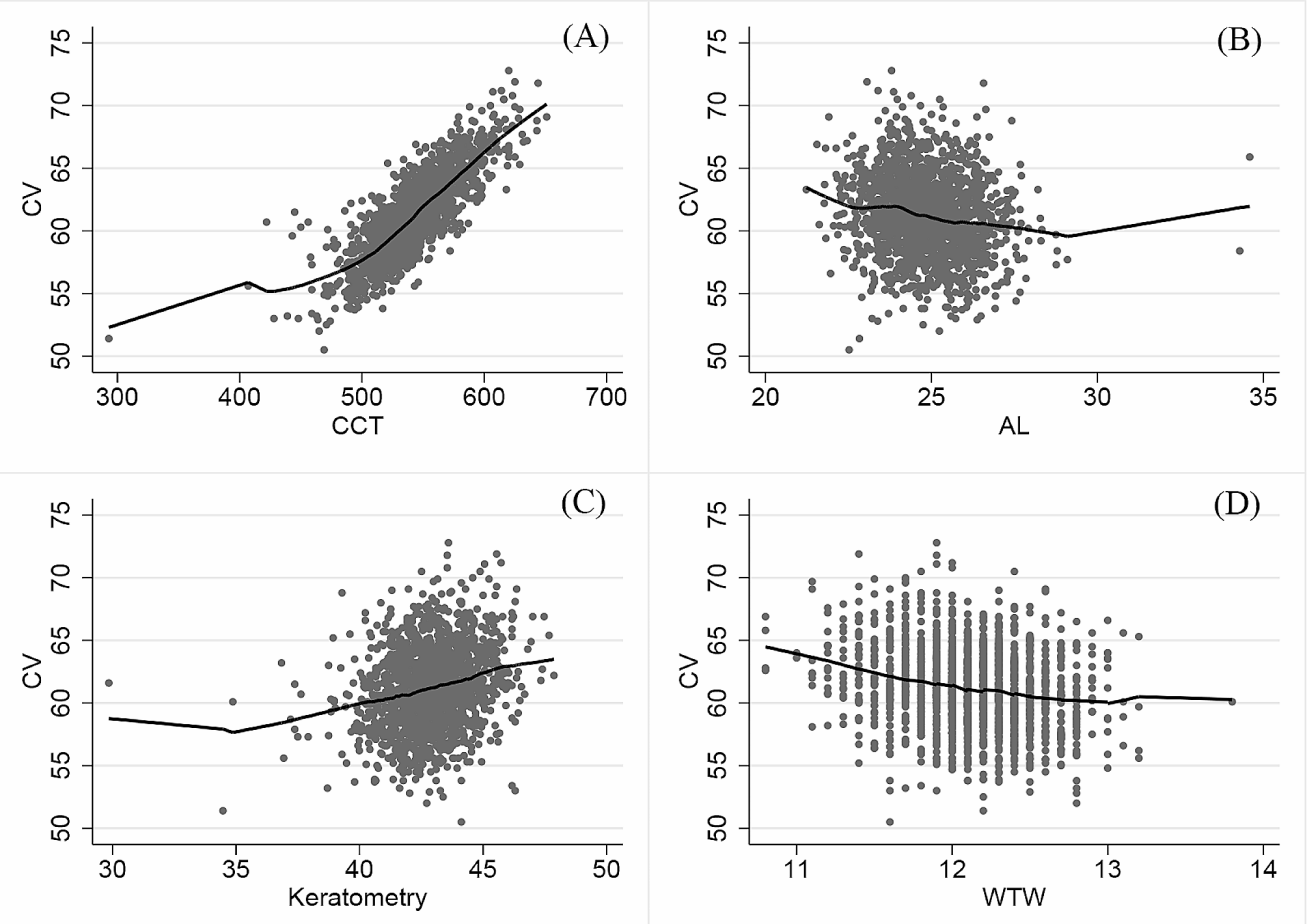
Lowess curves demonstrating the relationships between corneal volume and **(A)** central corneal thickness, **(B)** axial length, **(C)** keratometry, and **(D)** white to white distance. AL = axial length; WTW = white to white; CCT = central corneal thickness. All the subjects were included in the analysis. The solid black line is the fitting result of Lowess regression

Lowess curves were fitted to assess the relationships between CV and other ocular biometrical indices. (Fig. 2) The CV tended to increase with increasing CCT and keratometry. In contrast, CV had a continuous downward trend with AL and WTW distance

Table 3 shows the results of univariate and multivariate linear regression. Keratometry and CCT were positively correlated with CV, while AL and WTW distance had significant negative relationships with CV (p all < 0.001). Higher bIOP was associated with increased CV in the univariate linear regression model (β = 0.251, *p* < 0.001). However, in the multivariate linear regression model, CV was inversely associated with bIOP (β = -0.06, *p* < 0.001). CCT had the largest standardized regression coefficient (β = 0.199, *p* < 0.001). Moreover, we compared the CV between subjects with different refractive statuses, and no significant difference was observed.

**Table 3:** Association of corneal volume with other variables in univariate and stepwise multivariate linear regression models

Variable	Univariate					Multivariate			
	β coefficient	95%CI	*p*	β coefficient	95%CI		SRC	*p*	
Age, year	-0.090	(-0.259, 0.079)	0.30						
Sex	0.326	(-0.010, 0.663)	0.06						
Ethnicity	-0.012	(-0.086, 0.062)	0.74						
SE, D	0.024	(-0.041, 0.090)	0.47						
ACD, mm	-0.592	(-1.232, 0.048)	0.07						
AL, mm	-0.398	(-0.525, -0.270)	<0.001	-0.204	(-0.278, -0.130)		-0.077	<0.001	
Keratometry, diopter	0.483	(0.383, 0.583)	<0.001	0.422	(0.362, 0.481)		0.199	<0.001	
WTW, mm	-0.331	(-0.516, -0.145)	<0.001	-0.236	(-0.334, -0.139)		-0.061	<0.001	
CCT, micron	0.083	(0.080, 0.086)	<0.001	0.085	(0.083, 0.088)		0.842	<0.001	
bIOP, mmHg	0.251	(0.201, 0.301)	<0.001	-0.060	(-0.089, -0.032)		-0.057	<0.001	

CI = confidence interval; SRC = standardized regression coefficient; AL = axial length; SE = spherical equivalent; ACD = anterior chamber depth; WTW = white to white; CCT = central corneal thickness; bIOP = biomechanically corrected intraocular pressure

## Discussion

The present study investigated the normative values of CV and explored its associated factors among healthy young Chinese adults. As an abnormal CV is possibly suggestive of keratoconus or other forms of ectasia [5, 6], our findings may aid in preoperatively identifying patients with these eye conditions who are not suitable for refractive surgeries due to high risk of postoperative complications such as progressive ectasia [10, 11]. 

The mean CV for all eyes was 61.23 ± 3.22 mm^3^ in the present study. A mean CV of 60.1 ± 3.5 mm^3^ was reported by a study conducted among young Iranian adults with an average age of 29.3 years [12]. Hashemi et al. [4] found a mean CV of 57.92 mm^3^ in an elderly population older than 60, which was much smaller than our result. The age distribution of the participants could probably explain these discrepancies since aging was identified as a factor associated with CV reduction [4]. Therefore, it is necessary to establish age-specific standards to better differentiate normal corneas from abnormal corneas.

Compared with the Han ethnic group, we found significantly smaller CVs in the Yi ethnic group. Considerable discrepancies in various aspects between the two ethnic groups, such as lifestyle, culture and religion, could have contributed to the difference in CV. Thus, the CV may be related to ethnicity to some extent. However, there was no significant difference in CV between Han adults and the other minorities, which could be because the small sample of other minorities (fewer than 100 people per ethnic group) has limited the statistical power.

Based on the Lowess curves and the results of linear regression, CCT was found to be positively associated with CV. The study by Hashemi et al. [4] reported similar findings among elderly individuals, in which the decrease in CV accompanying thinning corneal thickness was considered age-related, and the correlation was speculated to be the result of increased corneal stiffness with age. However, the association between CV and CCT in the present study could hardly be attributed to the physiological change during the aging process because the age gap between the participants was too small (mostly aged between 18 and 20). The underlying mechanism is still unclear. Interestingly, except for CV, CCT was the only metric in which a significant difference was observed between Han and Yi adults and had the largest standard regression coefficient. These findings further confirmed the strong correlation between CCT and CV.

AL and WTW distance were found to be significantly negatively associated with CV, which is consistent with previous findings [4, 7]. It has been proposed that in myopia patients with a longer AL, the decrease in CV may be attributed to the lower corneal thickness [7]. However, a study by Price et al. showed that there was no correlation between CCT and AL [13], and the issue remains controversial. Another possible explanation is that the WTW distance tends to be longer with axial elongation, and the cornea would be stretched with flatter keratometry [4, 14]. Consequently, the cornea would be of denser tissue, which contributes to the reduction in CV [4]. Further studies are warranted to prove the interrelationship between these ocular biometrical parameters.

There was a positive association between CV and bIOP in the univariate analysis, but the relationship was reversed after controlling for other biometrical parameters. The results suggest that increased bIOP may result in a decrease in CV. This may be due to the higher IOP increasing the stress on the corneal tissue and hence decreasing the CV with denser tissue. The study by Jin et al. [7] also reported that CV was smaller in corneas with high myopia, whereas we found no significant difference between the mild or moderate myopic group and the high myopic group. This may be because they defined high myopia as AL ≥ 26 mm, while we defined high myopia according to the SE.

To our knowledge, this is the first study to provide normal CV values among healthy young Chinese adults. However, there are some limitations of our study. First, the cross-sectional design of the study made us unable to detect corneal changes with age. Second, there may be selection bias because the participants were all students from the same university, and young adults of the same age not attending school were not included. Third, myopia was diagnosed using noncycloplegic refraction, which may not be accurate.

## Conclusions

In conclusion, our study investigated the distribution of CV and explored its related factors among healthy young Chinese adults. Flatter keratometry values and thinner CCT were significantly associated with smaller CV. AL, WTW distance and bIOP had a significant inverse association with CV. The findings could probably be used as references for identifying suspect patients with corneal ectasia in clinical settings. Furthermore, the correlation of the CVs measured by Pentacam with those calculated using sim K and Q values is worthy of investigation in the future studies. In order to improve the sensitivity to detect early ectatic changes, it is also of great interest to explore how posterior cornea indices relate to the CV. Additional studies are needed to investigate these issues.

### Electronic supplementary material

Below is the link to the electronic supplementary material.

Supplementary Material 1

## Data Availability

The datasets generated and/or analyzed during the current study are not publicly available due to the risk of individuals privacy being compromised but are available from the corresponding author on reasonable request.

## Abbreviations

CV: Corneal volume
AL: Axial length
ACD: Anterior chamber depth
WTW: White-to-white
CCT: Central corneal thickness
bIOP: Biomechanically corrected intraocular pressure
SE: Spherical equivalent
SD: Standard deviation
ANOVA: Analysis of variance
SW: Shapiro‒Wilk
LOWESS: Locally weighted scatterplot smoothing
