# Supplementary material for: Distribution and determinants of corneal volume among healthy young Chinese adults: a cross-sectional study
Source: BMC Ophthalmol. 2024 Feb 12;24:59. doi: 10.1186/s12886-024-03342-8 (PMC10860269; doi:10.1186/s12886-024-03342-8)
Supplement: Supplementary file 1 — Supplementary Material 1 [file 12886_2024_3342_MOESM1_ESM.docx]

**Supplemental material**

**Supplemental Table S1. The distribution of corneal volume stratified by ethnicity.**

| **Ethnic group** | **n (%)** | **Mean ± SD** | ***P* value** |
| --- | --- | --- | --- |
| Han | 1239 (75.32) | 61.31 ± 3.24 |  |
| Yi | 151(9.18) | 60.49 ± 3.20 | .003* |
| Bai | 49(2.98) | 61.07 ± 2.85 | .60 |
| Dai | 18(1.09) | 62.37 ± 2.73 | .17 |
| Zhuang | 35(2.13) | 61.29 ± 3.15 | .97 |
| Miao | 24(1.46) | 61.05 ± 3.29 | .70 |
| Nakhi | 3(0.18) | 61.63 ± 5.05 | .86 |
| Other minorities | 126(7.66) | 61.20 ± 3.19 | .71 |

* *P* <.05 in comparison with Han adults; SD = standard deviation.

Analysis of variance (ANOVA) was used to make comparisons between all groups (*F* = 1.619, *P* = .125), which was followed by the LSD multiple comparison test.
